# 

**Published:** 1841-04

**Authors:** 


### CONTENTS

OF NO. IV.

### ORIGINAL COMMUNICATIONS.

### ESSAYS AND CASES.

ART. I.—A Memoir on Obliteration of the Bronchiæ: By A. C. REYNAUD, M. D., formerly resident in the Hospital; late Chief of Clinical Medicine of the Paris Faculty; Physician at Puy, &c. Translated for the Western Journal of Medicine and Surgery: By JOHN A. WARDER, M. D., of Cincinnati. - - - - - - 241

ART. II.—A Case of Triplets: By A. H. BUCHANAN, M. D., of Columbia, Tennessee. - - - - - - - 253

### REVIEWS.

ART. III.—Medical and Physiological Commentaries: By MARTYN PAINE, M. D. A. M. - - - - - - - 257

ART. IV.—Researches on the Pathology and Treatment of some of the most Important Diseases of Women: By ROBERT LEE, M. D., F. R. S., Physician-Accoucheur to the British Lying-in Hospital and the St. Mary-le-Bone Infirmary; Lecturer on Midwifery in the school of Webb street; London, 1833. pp. 220. - - - - 271

SELECTIONS FROM AMERICAN AND FOREIGN JOURNALS.

Sulphate of Quinine in Enlargement of the Spleen, and in Dropsies after Agues. - - - - - - - - - - 305
Cold Affusion in Hooping-cough. - - - - - - - 306
Traumatic Tetanus, successfully treated by Tobacco Enemas. - 308
Irritation of a Branch of the Trigeminus Nerve at its central extremity: By Dr. BUDGE, of Altenkirchen. - - - - 310
A Case of Posthumous Variola: By Dr. CHANSAREL - - - 314

ORIGINAL INTELLIGENCE.

Medicine in Dublin: By M. L. LINTON; M. D. - - - - 315
Axillary Aneurism. - - - - - - - - - 320
Death of Sir Astley Cooper. - - - - - - - 320

